# Association between the rs2106261 polymorphism in the zinc finger homeobox 3 gene and risk of atrial fibrillation: Evidence from a PRISMA-compliant meta-analysis

**DOI:** 10.1097/MD.0000000000027749

**Published:** 2021-12-10

**Authors:** Yue Wei, Lingjie Wang, Changjian Lin, Yun Xie, Yangyang Bao, Qingzhi Luo, Ning Zhang

**Affiliations:** Department of Cardiovascular Medicine, Ruijin Hospital, Shanghai Jiao Tong University School of Medicine, Shanghai, China.

**Keywords:** atrial fibrillation, rs2106261, single-nucleotide polymorphism, zinc finger homeobox 3

## Abstract

**Introduction:**

Previous genome-wide studies have identified an association between the rs2106261 single-nucleotide polymorphism (SNP) in the zinc finger homeobox 3 (ZFHX3) gene and an increased risk of atrial fibrillation (AF). However, this association remains controversial, since conflicting results have been reported in previous studies. We aimed to investigate the association between the ZFHX3 rs2106261 polymorphism and susceptibility to AF.

**Methods:**

A comprehensive literature search, of articles written in either English or Chinese, was conducted on various databases, including PubMed, Embase, Web of Science, the Cochrane library, Wan Fang, and CNKI, for studies performed up to August 1, 2020. Data were abstracted and pooled using Stata 14.0 software. A meta-analysis was performed on all selected studies based on ZFHX3 rs2106261 polymorphism genotypes.

**Results:**

Nine studies, including 10,107 cases and 58,663 controls, were analyzed in the meta-analysis. In the overall population, a significant association was found between AF and the T-allelic ZFHX 3 rs2106261 SNP (odds ratio [OR] = 1.32, 95% confidence interval [CI] 1.19–1.46). In subgroup analysis, a significant association between the T-allele of rs7193343 and risk of AF in Caucasian (OR = 1.23, 95% CI 1.10–1.37) and Asian subgroups (OR = 1.58, 95% CI 1.32–1.89) was observed. However, no statistically significant association was found in African populations (OR = 1.06, 95% CI 0.95–1.19).

**Conclusion:**

The genetic variant rs2106261 SNP is associated with susceptibility to AF in Caucasian and Asian individuals, with Asian samples showing a stronger association. However, based on the current evidence, no association was found in African samples. Future studies, with larger sample sizes and multiple ethnicities, are still necessary.

## 1 Introduction

Atrial fibrillation (AF) is one of the most common types of cardiac arrhythmias observed in clinical practice, affecting over 33.5 million individuals globally and showing an increase in prevalence.^[1]^ AF is associated with stroke and heart failure, causing substantial economic burden to patients’ families and society at large.^[2,3]^

Several factors associated with increased susceptibility to AF have been identified. These include age, male gender, obesity, valvular heart disease, hypertension, myocardial infarction, and family history.^[4,5]^ In recent years, genetic predisposition has been identified as an additional risk factor for AF, as genome-wide association studies (GWAS) have identified a great number of genetic variants in specific genes associated with AF.^[6]^ While the underlying mechanism responsible for these associations remains to be elucidated, it is likely that risk haplotypes affect the expression of neighboring genes.

Previous GWAS have shown that the single-nucleotide polymorphism (SNP) rs2106261 in the zinc finger homeobox 3 (ZFHX3) gene at chromosome 16q22 is strongly associated with susceptibility to AF.^[7]^ The association between allele T at rs2106261 and AF has been replicated in several populations. However, conflicting results have been reported, showing that the presence of the T risk allele may not be a specific risk factor for AF. To address these conflicting results, the current meta-analysis was conducted to further evaluate the associations between the rs2106261 SNP and AF risk.

## 2 Methods

The present review was conducted in accordance with the Preferred Reporting Items for Systematic Reviews and Meta-Analyses guidelines. The protocol for our study was registered in the International Prospective Register of Systematic Reviews, with the registration number CRD42018106938 (available from: https://www.crd.york.ac.uk/prospero/display_record.php?RecordID=106938).

### 2.1 Literature search

Scientific databases, such as PubMed, EMBASE, web of science, the Cochrane library, Wan Fang, and CNKI, were searched for studies related to rs2106261 ZFHX3 polymorphism and AF. The Medical Subject Heading terms “AF,” “zinc finger homeobox 3,” “genetic polymorphism,” and the free-text words “ZFHX3” or “rs2106261” were combined. The reference lists of all eligible studies were checked for identification of further relevant research.

### 2.2 Study selection and quality assessment

Studies that were included in our meta-analysis met the following criteria:

1. published articles from peer-reviewed medical journals;
2. case-control, nested case-control, cross-sectional design, or cohort studies;
3. investigation of the association of the rs2106261 ZFHX3 polymorphism and susceptibility to AF; and
4. sufficient data provided to extract odds ratios (ORs) and 95% confidence intervals (CIs).

Unpublished studies, review articles, abstracts-only articles, case reports, editorials, and studies that did not contain adequate outcomes of interest were excluded from the current analysis.

Two investigators (W.Y. and Z.N.) independently screened each citation for inclusion. Two reviewers (W.Y. and Z.N.) independently reviewed the full-text of articles to determine eligibility. Discrepancies were resolved by discussion with the entire research team. The following data were extracted from the included studies: the first author, publication year, country where work was conducted, number of patients/control individuals, average age/gender distribution of patients/control individuals, source of controls, genotyping method, genotype distribution, and data necessary to conduct meta-analysis. Attempts to contact the original authors for detailed information were made if the data were incomplete or missing in the publication. Quality of prospective or case-control studies was evaluated according to the nine-point Newcastle-Ottawa Scale, and quality of cross-sectional studies was evaluated according to the Agency for Healthcare Research and Quality standards.

### 2.3 Statistical analysis

Statistical analysis was performed with Stata 14 (Stata Corporation, College Station, TX). Strength of the association between the rs2106261 ZFHX3 polymorphism and susceptibility to AF was assessed by combining OR and CIs. Cochran's Q statistic and the *I*^2^ index were used to calculate heterogeneity across all included studies. If *P* < .1 or *I*^2^ > 50%, a random-effects model was used to estimate the pooled OR (DerSimonian and Laird methods), due to the presence of significant heterogeneity^[8]^; otherwise, a fixed-effects model was used (the Mantel-Haenszel method).^[9]^

Sensitivity analysis was performed by calculating ORs repeatedly with omission of each study to check the consistency of the overall estimated effect. Publication bias was conducted both visually (via funnel plot) and statistically (via Begg and Egger bias tests). All statistical testing was two-tailed with a statistical significance set at *P* < .05.

## 3 Results

### 3.1 Literature search

**Figure 1:**
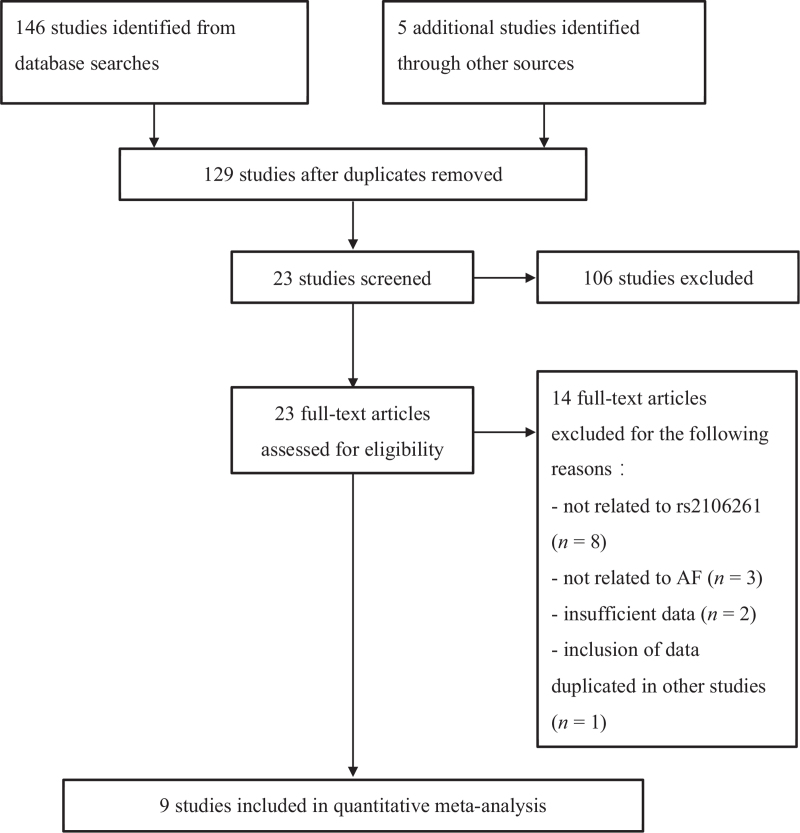
Flowchart of the systematic literature research for the meta-analysis.

The electronic search yielded 151 potentially relevant abstracts. After removing duplicates and ineligible abstracts, we reviewed the full text of 23 reports. Of these reports, 14 were excluded for not meeting inclusion criteria. Consequently, nine studies were finally selected in this meta-analysis.^[7,10–17]^ The results of the search and selection process are shown in Figure 1.

### 3.2 Study characteristics

Table 1 summarizes the characteristics of the included studies. A total of 15 comparisons were involved in the current meta-analysis, containing 10,107 cases and 58,663 controls. One article as a brief communication was a multi-center cohort study including 6 comparisons,^[7]^ and another article included 2 comparisons.^[15]^ The sample sizes ranged from 743 to 11,639. The ethnicities of the included comparisons were Caucasian (n = 7), Asian (n = 5), and African (n = 3). Genotypic distributions in controls from most selected studies conformed to Hardy Weinberg Equilibrium. Quality of included studies was considered good according to Newcastle-Ottawa Scale and Agency for Healthcare Research and Quality standards. Results are shown as supplementary data (see Supplemental Digital Content Table S1 and S2, supplementary content, which illustrates the quality of studies).

**Table 1:** Characteristics of included studies.

				Age		Gender (M/F)					
Comparison	Ethnicity	Case	Control	Case	Control	Case	Control	Source of control	MAF	Genotyping method	HWE
Benjamin 2009 (AFNET)	Caucasian	2145	4073	61.0	49.2	1,564/581	2004/2069	Population	19.0%	Sequenom	NA
Benjamin 2009 (AGE)	Caucasian	379	5298	78.2	76.5	148/231	1971/3327	Population	17.4%	Illumina	Yes
Benjamin 2009 (ARIC White)	Caucasian	731	7355	67.0	56.0	NA	NA	Population	17.4%	Affymetrix	Yes
Benjamin 2009 (CHS White)	Caucasian	829	5639	81.2	70.9	324/505	2188/3451	Population	17.4%	Illumina	Yes
Benjamin 2009 (FHS)	Caucasian	623	8025	74.3	64.4	280/343	3507/4518	Population	17.4%	Affymetrix	Yes
Benjamin 2009 (RS)	Caucasian	851	10,788	77.7	68.6	346/505	4348/6440	Population	17.0%	Illumina	Yes
Li 2011 China	Asian	650	1447	58.4	59.7	398/252	902/545	Population	34.9%	Wizard	Yes
Olesen 2012 Denmark	Caucasian	209	534	40.0	65.0	171/38	278/256	Hospital	22.0%	Taqman	Yes
Perez 2013 US	African	558	7561	63.4	61.5	0/558	0/7,561	Population	25.3%	Affymetrix	Yes
Liu 2014 China	Asian	597	996	58.4	59.0	397/200	674/322	Hospital	36.5%	Sequenom	Yes
Choi 2015 Korean	Asian	1068	1068	57.5	57.5	797/271	797/271	Hospital	39.5%	Taqman	Yes
Roberts 2016 (CHS Black)	African	71	740	NA	NA	NA	NA	Population	26.4%	Illumina	Yes
Roberts 2016 (ARIC Black)	African	582	2531	NA	NA	NA	NA	Population	26.1%	Affymetrix	Yes
Zaw 2017 Japan	Asian	452	1981	82.8	80.1	255/197	1076/905	Hospital	30.1%	Illumina	Yes
Tomomori 2018 Japan	Asian	362	627	62.4	53.0	268/313	313/94	Hospital	33.3%	Taqman	Yes

### 3.3 Association of rs2106261 polymorphism of the ZFHX3 gene with the risk of AF

**Figure 2:**
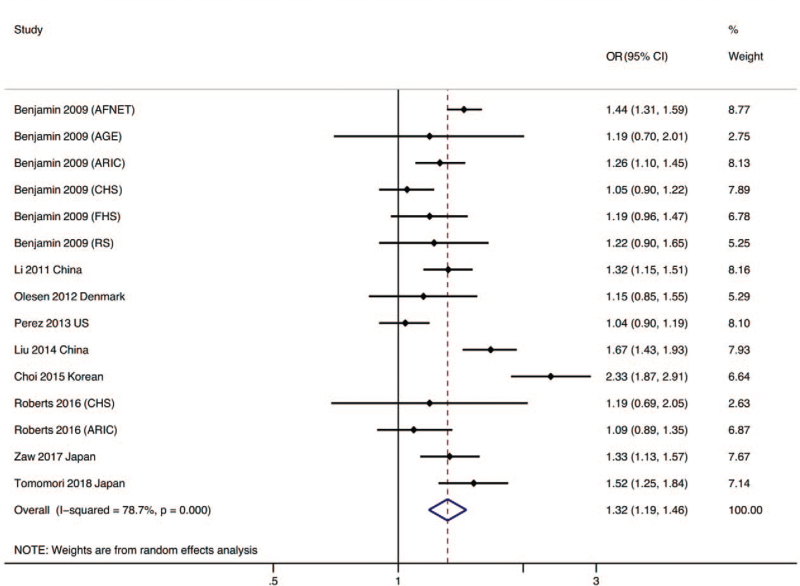
Forest plot for overall studies examining the association between ZFHX3 rs2106261 SNP and AF. AFNET = German AF Network, AGES = Age, Gene/Environment Susceptibility Study, ARIC = Atherosclerosis Risk in Communities Study, CHS = Cardiovascular Health Study, CI = confidence interval, FHS = Framingham Heart Study, OR = odds ratio, RS = Rotterdam Study.

A random-effects model was used to analyze data from all selected studies, which generated a combined OR of 1.32 for risk allele (95% CI 1.19–1.46, *P* < .001; *I*^2^ = 78.7%, *P* for heterogeneity (*P*_het_) < .001). These results indicated that there was a statistically significant association between the rs2106261 SNP and AF (see Fig. 2).

**Figure 3:**
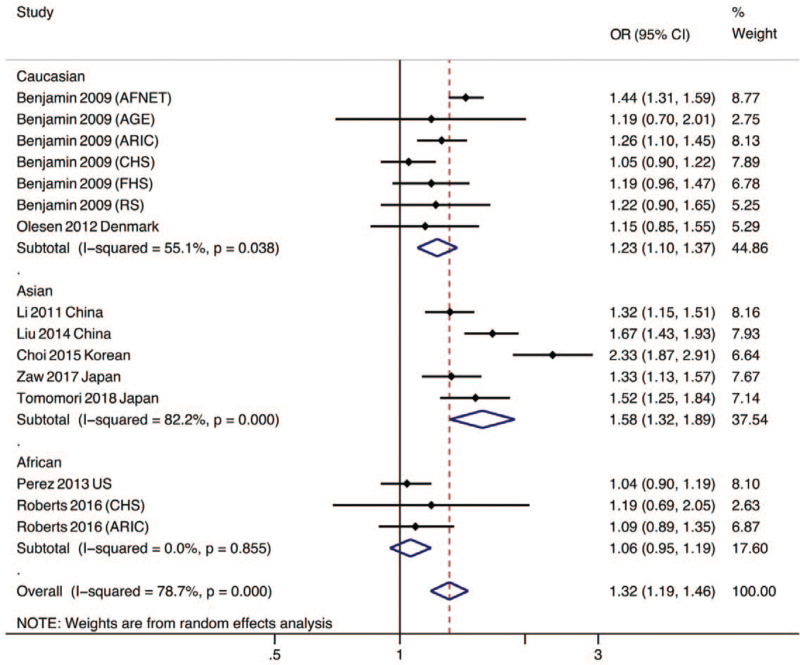
Subgroup analysis by ethnicity of the association between the ZFHX3 rs2106261 SNP and AF. AFNET = German AF Network, AGES = Age, Gene/Environment Susceptibility Study, ARIC = Atherosclerosis Risk in Communities Study, CHS = Cardiovascular Health Study, CI = confidence interval, FHS = Framingham Heart Study, OR = odds ratio, RS = Rotterdam Study.

In the subgroup analysis by ethnicity, significant associations between rs2106261 and AF were observed in Asian (OR = 1.58, 95% CI 1.32–1.89, *P* < .001; *I*^2^ = 82.2%, *P*_het_ < .001) and Caucasian (OR = 1.23, 95%CI 1.10–1.37, *P* < .001; *I*^2^ = 55.1%, *P*_het_ < .001) samples. However, in African samples, no significant association was observed (OR = 1.06, 95% CI 0.95–1.19, *P* = .31; *I*^2^ = 0%, *P*_het_ = .86; see Fig. 3).

### 3.4 Sensitivity analysis

**Figure 4:**
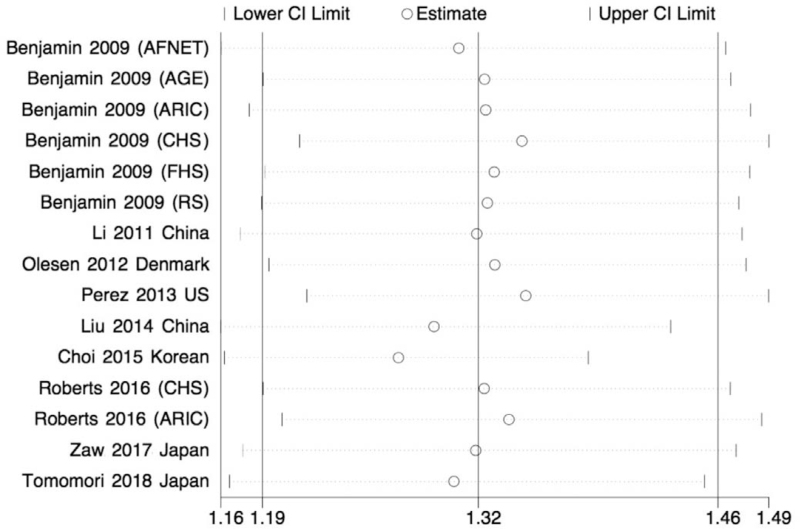
Sensitivity analysis of all analyzed studies. CI = confidence interval.

Sensitivity analyses were conducted to discover whether the omission of each study quantitatively altered the pooled ORs. As shown in Figure 4, it was revealed that no single study altered the stability of the overall results, providing evidence of an increased risk of the rs2106261 ZFHX3 SNP to AF susceptibility.

### 3.5 Publication bias

**Figure 5:**
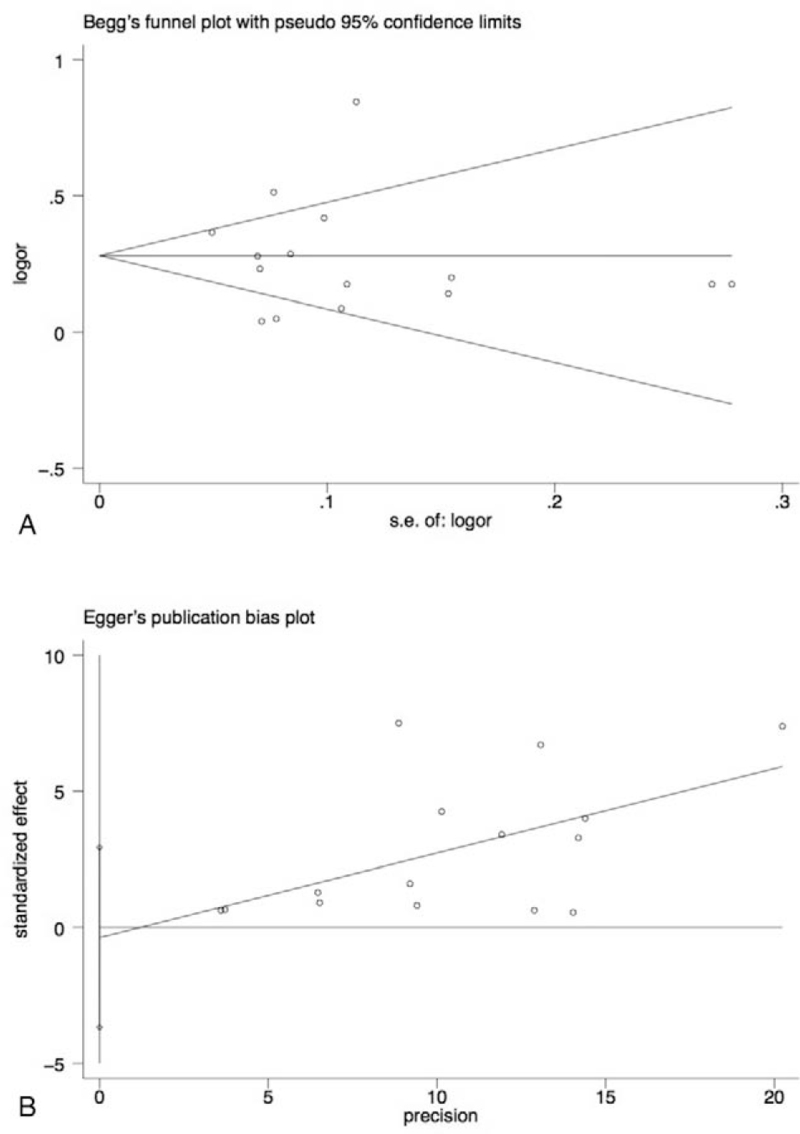
Funnel plots of all analyzed studies. (A) Begg funnel plot. (B) Egger funnel plot.

Begg funnel plot and Egger test were performed to estimate publication bias. Begg funnel plot showed an approximately symmetrical shape (*P* = .92), and no publication bias was observed in the Egger test (*P* = .81; see Fig. 5).

## 4 Discussion

In recent years, the association between genetic variants and AF has become increasingly apparent since GWAS have identified a number of SNPs associated with AF. In our analysis, a robust association with AF was observed for the rs2106261 SNP, providing solid evidence for a genetic basis of AF pathophysiology.

### 4.1 Role of ZFHX3 in the pathophysiology of AF

The ZFHX3 gene is located on chromosome 16q^22.2–22.3^ and encodes AT motif binding factor 1 (ATBF1), which is a transcription factor with multiple homeodomains and zinc finger motifs. ATBF1 has been reported to inhibit cell proliferation, thus functioning as a tumor suppressor in several cancers. ZFHX3 is widely expressed in many tissues including the heart. In a rapid pace-induced porcine AF model, where significant structural and inflammatory changes were noted in the atria, the expression of signal transducer and activator of transcription 3 (STAT3) was up-regulated, and nuclear translocation and binding to STAT3 cis-elements of the target were increased, suggesting that STAT3 plays a critical role in the pathogenesis of AF.^[18]^ ZFHX3 interacts with the terminal end of the protein inhibitor of activated STAT 3, which is a specific inhibitor through binding to activated tyrosine-phosphorylated STAT3 dimers, subsequently suppressing STAT3-mediated signaling pathway.^[19,20]^ Additionally, knockdown of ZFHX3 has been shown to enhance the activation of STAT3, increasing the expression of sarcoplasmic/endoplasmic reticulum calcium ATPase 2a, ryanodine receptors, and several potassium channels, leading to dysregulation of calcium homeostasis and an increase in atrial arrhythmogenesis. Overexpression of ZFHX3 reversed the above effects. Taken together, the above results indicate that inhibition of ZFHX3 might contribute to AF.^[21]^ Moreover, ZFHX3 has been observed to regulate protein sumoylation in different biological processes, through which it may play an additional role in the pathophysiology of AF by regulating certain related proteins.^[22]^ Finally, ATBF1 could interact with Runt domain transcription factor 3, with the complex translocated to the nucleus upon activation of the transforming growth factor-beta signaling pathway, thus transactivating pro-fibrotic genes, which can contribute to atrial remodeling and atrial fibrosis.^[23,24]^ However, the role of ZFHX3 in the pathophysiology of AF has not been fully elucidated, and further fundamental studies are still warranted.

### 4.2 Relationship between rs2106261 and AF

The rs2106261 SNP of the ZFHX3 gene was first identified as a locus for AF by GWAS. This risk variant was first discovered to be significantly associated with AF in 5 community-based cohorts in the United States and Iceland, with results replicated in a Germany cohort.^[25]^ Soon afterward, a study by Li et al^[10]^ showed that the minor allele of rs2106261 was related to AF susceptibility in a Chinese Han population, expanding the association between ZFHX3 and AF in individuals of non-European ancestry. This result remained significant when only isolated AF was included in the analysis, suggesting that ZFHX3 gene might play an important role in the specific pathophysiology of AF. Later, the relationship between rs2106261 and AF in Korean and Japanese populations was identified and replicated, respectively,^[16,14]^ therefore confirming that the risk allele of rs2106261 was associated with AF in the entire East-Asian population. Overall, rs2106261 is associated with susceptibility of AF in large populations, including individuals of East-Asian and European descent.

### 4.3 Role of ethnicity in the association between rs2106261 and AF

Ethnicity affects not only minor allele frequency (MAF) but also the association of the minor allele and susceptibility to AF. In our study, it was found that the distribution of the rs2106261 SNP genotype in ZFHX3 gene differs among populations. The MAF of rs2106261 ranges from 17% to 22% in Caucasian populations and has been found to be approximately 25% in African populations. However, the MAF in Asian populations was found to be above 30%. Similarly, the MAF of rs7193343, another SNP of the ZFHX3 gene, is observed more frequently in individuals of Chinese descent than of individuals in a Caucasian population.^[26]^ Taken together, these results suggest that ethnicity is of great importance in polymorphism distribution of ZFHX3 gene. Moreover, the association of rs2106261 risk allele and increased risk of AF is significant in Asian and European populations, but not in African populations. A previous meta-analysis observed that rs7193343 was associated with AF in Caucasian populations, but not in an Asian population. Potential reasons for these discrepancies are as follows:

1. inconsistency of genetic background between ethnicities^[27]^;
2. different lifestyle and/or diet practiced by various ethnicities^[28]^; and
3. different gene-environment interactions under different regional environmental conditions.^[29]^

Further genetic studies are necessary to better understand the precise underlying mechanisms responsible for these differences.

### 4.4 The ZFHX3 polymorphism and outcomes of AF ablation

Catheter ablation, which is widely used in clinical practice, is recommended as the treatment of drug-resistant, symptomatic AF. In recent years, studies have shown that several SNPs associated with AF could predict AF recurrence after AF ablation. A study by Husser et al^[30]^ was the first study that tested the effect of 2 SNPs (rs2200733 and rs10033464) on chromosome 4q25, which had been previously associated with AF. The study examined short-term and long-term outcomes after AF catheter ablation and found that the presence of either SNP increased the risk for both early and late recurrence of AF. A meta-analysis validated the fact that the rs2200733 polymorphism increases the risk of AF recurrence after catheter ablation.^[31]^ As for the rs2106261 polymorphism, it has been reported that it was not associated with AF recurrence after radiofrequency ablation^[14,32]^ or after cryoballoon ablation.^[33]^ In these studies, both paroxysmal and persistent AF patients were enrolled, and strategies for ablation were multifarious. However, Tomomori et al^[17]^ revealed that the minor allele of rs2106261 is associated with long-term recurrence in paroxysmal AF in patients undergoing pulmonary vein isolation. In addition, Parker found that the minor allele of rs2106261 was a marker of a good response to radiofrequency ablation in long-standing AF. One possible explanation is that ZFHX3 positively regulates expression of paired-like homeodomain 2c, which is necessary to differentiate pulmonary mesenchyme into myocardium, expanding by proliferation to form the pulmonary myocardial sleeve. Therefore, the minor allele of the rs2106261 ZFHX3 polymorphism might modulate the expression of paired-like homeodomain 2c, promoting the proliferation of the myocardial sleeve around the branches of the pulmonary vein, increasing automaticity, which then serves as an AF trigger.^[34,35]^ Under this circumstance, AF is related to the pulmonary vein and thus could be cured by pulmonary vein isolation. Further studies are still warranted to determine the association of rs2106261 polymorphism and outcome of AF ablation and its potential underlying mechanism.

## 5 Limitations

The current study has a number of limitations. First, in our analysis, significant heterogeneity was observed in the overall population. After subgroup analysis was performed, no heterogeneity was found in the African population, while a trend of decreasing heterogeneity was seen in the European population, and heterogeneity remained significant in the Asian population. Nonetheless, we used sensitivity analysis methods in the random-effects model and found no difference in risk ratio or its 95% confidence interval. The heterogeneity may have been caused by

1. variation in participant characteristics (e.g., age, sex, general condition, medical history, and habits) existed between studies;
2. type, duration, and etiology of AF were different among studies;
3. underlying haplotype structure was different between study populations; or
4. study-specific precision was low in some of the studies included.

Second, all of the studies were published in English. Studies in other languages or unpublished studies were not included in this meta-analysis, which may have resulted in selection and/or publication bias. Third, the genotyping data of rs7193343 SNPs were insufficient, so we were unable to conduct meta-analysis based on the limited data available from the studies analyzed. Fourth, in the African population subgroup, the total sample size of AF patients was much less than that observed in the other 2 populations. More studies with larger sample sizes are needed to confirm the current conclusions. Fifth, other factors have also been associated with AF risk (e.g., age, sex, hypertension, coronary heart disease, and family history). The results would be more precise if individual data were adjusted controlling for these variables.

## 6 Conclusion

The genetic variant rs2106261 SNP is associated with susceptibility to AF in Caucasian and Asian populations, although this association is more significant in Asian populations. However, the current data show no association in African populations. Further studies with larger sample sizes and multiple ethnicities are still warranted in the future.

## Acknowledgments

We thank LetPub (www.letpub.com) for its linguistic assistance during the preparation of this manuscript.

## Author contributions

**Conceptualization:** Yue Wei, Lingjie Wang.

**Data curation:** Yue Wei, Lingjie Wang, Ning Zhang.

**Formal analysis:** Yue Wei, Lingjie Wang, Ning Zhang.

**Funding acquisition:** Yangyang Bao, Ning Zhang.

**Investigation:** Yue Wei, Lingjie Wang.

**Methodology:** Yue Wei, Lingjie Wang.

**Project administration:** Yue Wei, Lingjie Wang, Yangyang Bao.

**Resources:** Yue Wei, Changjian Lin, Yun Xie, Yangyang Bao, Qingzhi Luo.

**Software:** Yue Wei, Changjian Lin, Yun Xie, Yangyang Bao, Qingzhi Luo.

**Supervision:** Yun Xie, Yangyang Bao, Qingzhi Luo, Ning Zhang.

**Validation:** Changjian Lin, Yun Xie, Yangyang Bao, Qingzhi Luo.

**Visualization:** Changjian Lin, Yun Xie, Yangyang Bao, Qingzhi Luo.

**Writing – original draft:** Yue Wei, Lingjie Wang.

**Writing – review & editing:** Changjian Lin, Yun Xie, Yangyang Bao, Ning Zhang.

## Supplementary Material

Supplemental Digital Content

## Supplementary Material

Supplemental Digital Content
