# Supplementary material for: Association between the rs2106261 polymorphism in the zinc finger homeobox 3 gene and risk of atrial fibrillation: Evidence from a PRISMA-compliant meta-analysis
Source: Medicine (Baltimore). 2021 Dec 10;100(49):e27749. doi: 10.1097/MD.0000000000027749 (PMC8663867; doi:10.1097/MD.0000000000027749)
Supplement: Supplemental Digital Content [file medi-100-e27749-s001.docx]

Table S1. Quality assessment of prospective cohort or case-control studies according to the Newcastle-Ottawa Scale.

|  | Selection | Comparability | Exposure |
| --- | --- | --- | --- |
| Benjamin 2009 (CHS White) | ★★★★ | ★★ | ★★★ |
| Benjamin 2009 (AFNET) | ★★★★ | ★★ | ★★★ |
| Li 2011 | ★★★ | ★★ | ★★★ |
| Olesen 2012 | ★★★ | ★★ | ★★★ |
| Perez 2013 | ★★★ | ★ | ★★★ |
| Liu 2014 | ★★★ | ★★ | ★★★ |
| Choi 2015 | ★★★ | ★★ | ★★★ |
| Roberts 2016 (CHS Black) | ★★★★ | ★★ | ★★★ |
| Zaw 2017 | ★★★ | ★★ | ★★★ |
| Tomomori 2018 | ★★★ | ★★ | ★★★ |
