# Supplementary material for: Association between the rs2106261 polymorphism in the zinc finger homeobox 3 gene and risk of atrial fibrillation: Evidence from a PRISMA-compliant meta-analysis
Source: Medicine (Baltimore). 2021 Dec 10;100(49):e27749. doi: 10.1097/MD.0000000000027749 (PMC8663867; doi:10.1097/MD.0000000000027749)
Supplement: Supplemental Digital Content [file medi-100-e27749-s002.docx]

Table S2. Quality assessment of cross-sectional studies according to the Agency for Healthcare Research and Quality standards.

Y: Yes, N: No, and UC: Unclear

| Item | Benjamin  (AGE) | Benjamin  (ARIC White) | Benjamin  (FHS) | Benjamin  (RS) | Roberts  (ARIC Black) |
| --- | --- | --- | --- | --- | --- |
| 1) Define the source of information (survey, record review) | Y | Y | Y | Y | Y |
| 2) List inclusion and exclusion criteria for exposed and unexposed subjects (cases and controls) or refer to previous publications | Y | Y | Y | Y | Y |
| 3) Indicate time period used for identifying patients | Y | Y | Y | Y | Y |
| 4) Indicate whether or not subjects were consecutive if not population-based | Y | Y | Y | Y | Y |
| 5) Indicate if evaluators of subjective components of the study were masked to other aspects of the status of the participants | UC | UC | UC | UC | UC |
| 6) Describe any assessments undertaken for quality assurance purposes (e.g., test/retest of primary outcome measurements) | UC | Y | Y | Y | Y |
| 7) Explain any patient exclusions from analysis | N | Y | N | N | Y |
| 8) Describe how confounding was assessed and/or controlled | UC | UC | UC | UC | UC |
| 9) If applicable, explain how missing data were handled in the analysis | UC | UC | UC | UC | UC |
| 10) Summarize patient response rates and completeness of data collection | Y | Y | Y | Y | Y |
| 11) Clarify what follow-up, if any, was expected and the percentage of patients for which incomplete data or follow-up was obtained | UC | Y | Y | Y | Y |
